# Editorial: Interconnected lives: experiences and resilience of transnational families in a shifting global landscape

**DOI:** 10.3389/fsoc.2026.1776469

**Published:** 2026-01-27

**Authors:** Anastassia Zabrodskaja

**Affiliations:** Baltic Film, Media and Arts School, Tallinn University, Tallinn, Estonia

**Keywords:** collective resilience, familial relationships, global mobility, global wellbeing, interdisciplinary research, migration dynamics, third culture kid, transnational families

## 1 Introduction

Contemporary transnational families are shaped by intersecting processes of globalization, migration governance, economic inequality, and sociopolitical change. Rather than representing temporary or deficient arrangements, transnational families constitute enduring social formations through which care, belonging, identity, and responsibility are negotiated across borders. This Research Topic brings together interdisciplinary contributions that critically examine how family life is lived, sustained, and transformed under conditions of mobility and separation. Moving beyond economistic, deficit-oriented, and normatively bounded frameworks, the Research Topic foregrounds relational, emotional, linguistic, and generational dimensions of transnational family life. The articles span diverse geographical contexts and methodological approaches, addressing both structural inequalities and everyday practices through which families navigate education, caregiving, language, and belonging. Collectively, they challenge static notions of family, presence, and integration by conceptualizing transnational families as dynamic social fields shaped by power relations and uneven access to resources.

## 2 Theoretical contributions

The contributions significantly advance theorizing on transnational families by shifting attention from economistic and deficit-oriented perspectives toward relational, emotional, and intersectional approaches. Together, the articles conceptualize transnational family life as a dynamic social field shaped by power relations, gender norms, language hierarchies, generational positioning, and spatial inequalities, sustained across borders through everyday interactions, communication technologies, and emotional labor. Several contributions engage with the concept of ambiguous loss (Boss, 1999), illustrating how prolonged separation generates enduring emotional uncertainty that is neither fully resolvable nor uniformly experienced, particularly among transnational parents and children. Others mobilize Bourdieu's theory of social reproduction and capital (Bourdieu, 1986) to reveal how inequalities linked to class, education, birthplace, and language are reproduced across generations and institutional contexts, including settings often perceived as meritocratic, such as sport and higher education. The concept of Thirdness (Bhabha, 2004) further enriches the Research Topic by theorizing transnational families as sites of hybridity where identities and belonging are continuously negotiated. Several articles decenter adult-centric and moralizing assumptions by foregrounding children's, adolescents', Indigenous students', and marginalized migrants' perspectives, contributing to critical debates on agency, care, and resilience (James and Prout, 1997; Spyrou, 2018).

## 3 Methodological contributions

Methodologically, the Research Topic demonstrates the value of pluralistic, reflexive, and innovative research designs for capturing the complexity of transnational family lives. Qualitative approaches—including phenomenological interviews, ethnography, discourse analysis, and visual methods—are used to access emotional, relational, and symbolic dimensions of migration that often elude large-scale datasets. Phenomenological and Verstehen-oriented approaches enable nuanced understandings of lived experience, subjectivity, and meaning-making across borders (Schütz, 1967; van Manen, 2023). Visual and creative methods, including collage-based elicitation, illuminate affective dimensions of family life, memory, and identity that are difficult to articulate verbally (Harper, 2002; Pink, 2021). Large-scale quantitative and mixed-methods studies identify structural patterns related to wellbeing, social isolation, and adaptation.

## 4 Empirical and thematic contributions of the Research Topic

### 4.1 Structural inequality, marginality, and uneven life chances

Several contributions demonstrate how transnational and mobile lives are embedded in deeply unequal social, economic, and geopolitical structures, revealing how mobility both reproduces and reshapes inequality. Ahmad's analysis of children born into migrant rag-picking families exposes the intersection of poverty, discrimination, and institutional exclusion. Similarly, de Souza et al. show how regional inequalities within Brazil are reproduced through elite futsal careers, where birthplace functions as symbolic capital that advantages athletes from wealthier regions while rendering others structurally invisible. At the educational level, Guerra Ayala et al. and Zharkynbekova et al. highlight how linguistic hierarchies and national belonging shape students' inclusion and exclusion. Indigenous students in Peru and ethnic Kazakh repatriates in Kazakhstan encounter institutional environments that implicitly privilege dominant languages and cultural norms, producing social isolation or uneven adaptation.

### 4.2 Transnational care, emotional labor, and gendered responsibilities

A second cluster of articles critically examines transnational caregiving and emotional labor, with particular attention to gendered expectations and moral economies of care. Haider's study of Pakistani migrant fathers in Italy challenges economic-centered models of fatherhood by foregrounding emotional pain, moral obligation, and mediated intimacy, revealing how masculinity and care are renegotiated under conditions of prolonged separation. In contrast, Shamase and Sekaja and Domingo focus on women who remain physically closer to children—either as migrant mothers working abroad or as non-migrant mothers left behind—yet shoulder disproportionate emotional and practical responsibilities. Together, these studies complicate binary distinctions between “present” and “absent” parents by showing how caregiving and authority are continuously performed across distance.

### 4.3 Children's and young people's agency beyond adult-centered narratives

Several contributions decenter adult perspectives by foregrounding children's and young people's own evaluations of transnational family life. König et al. demonstrate that children in Poland do not passively internalize dominant moral discourses that stigmatize parental migration. Instead, they apply nuanced criteria—such as motives for migration, communication quality, and emotional support—when assessing what constitutes a “good childhood,” while asserting their right to information and participation in family decisions. Ahmad's and Guerra Ayala et al.'s findings highlight children's and students' aspirations, coping strategies, and resilience even under conditions of structural disadvantage. Collectively, these studies challenge deficit-oriented frameworks and contribute to a more differentiated understanding of agency that acknowledges constraints without denying young people's interpretive capacities.

### 4.4 Language, belonging, and transcultural family practices

Language emerges as a central analytical lens across several contributions, not only as a communicative tool but as a symbolic, emotional, and relational resource. Li-Gottwald, Bloch, Protassova and Yelenevskaya, and Li et al. demonstrate how language practices are embedded in everyday “doing family,” shaping intergenerational ties, identity formation, and belonging. These studies show that multilingualism is rarely a linear or harmonious process; instead, it involves negotiation, ambivalence, and unequal access to resources. Language learning and maintenance operate within broader power relations—between host and heritage languages, between generations, and between institutional expectations and family aspirations. By situating language within transnational family dynamics, these articles move beyond instrumental views of linguistic competence toward a relational understanding of language as lived experience.

### 4.5 Methodological innovation and conceptual expansion

Finally, the Research Topic advances the field methodologically and conceptually. Dillon and Ali's use of collage-based elicitation illustrates how visual and phenomenological methods can capture the affective and symbolic dimensions of transnational life that often remain inaccessible through conventional interviews. Barros et al.'s large-scale survey complements these qualitative insights by demonstrating how wellbeing emerges from the interaction of personal resources and contextual conditions, reinforcing the value of methodological pluralism.

## 5 Practical, policy, and pedagogical contributions

From a practical and policy-oriented perspective, the findings underscore the need for inclusive, intersectional, and family-sensitive frameworks that recognize transnational families as enduring social units. Access to education, healthcare, language training, labor markets, and social protection emerges as unevenly distributed and closely tied to migration status, gender, class, ethnicity, and regional inequality. The contributions challenge policy frameworks that privilege physical co-presence and nuclear family norms, instead calling for recognition of emotional labor, transnational caregiving, multilingual practices, and children's agency (Bryceson and Vuorela, 2002; Baldassar and Merla, 2014). Pedagogically, schools and universities function as critical sites of inclusion or exclusion for migrant, Indigenous, and transnational students. Language policies, pedagogical practices, and institutional cultures can either exacerbate marginalization or foster belonging and resilience. The findings reinforce the importance of culturally responsive pedagogy, language-sensitive instruction, and supportive learning environments that validate students' linguistic repertoires, cultural backgrounds, and lived realities (Cummins, 2000; Gay, 2018).

## 6 Directions for future research

While the contributions substantially advance scholarship on transnational families, they also point to several directions for future research. Greater use of longitudinal designs is needed to trace how family practices, identities, and inequalities evolve across life stages. Further attention to South–South migration, Indigenous mobility, and non-elite forms of transnationalism would address persistent gaps in the literature. Comparative and multi-sited research can illuminate how welfare regimes, education systems, and migration policies shape transnational family experiences across contexts. Continued methodological innovation—particularly participatory, visual, and child-centered approaches—remains essential for ensuring that marginalized voices are meaningfully centered in knowledge production.
